# Prevalence and Antimicrobial-Resistance of *Pseudomonas aeruginosa* in Swimming Pools and Hot Tubs

**DOI:** 10.3390/ijerph8020554

**Published:** 2011-02-18

**Authors:** Jonathan K. Lutz, Jiyoung Lee

**Affiliations:** 1 Division of Environmental Health Sciences, College of Public Health, The Ohio State University, Columbus, OI 43210, USA; E-Mail: jlutz@cph.osu.edu; 2 Department of Food Science & Technology, The Ohio State University, 375 Howlett Hall, 2001 Fyffe Ct. Columbus, OI 43210, USA

**Keywords:** *Pseudomonas aeruginosa*, Antimicrobial resistance, indoor recreational water, hot tub, swimming pool

## Abstract

*Pseudomonas aeruginosa* is an important opportunistic pathogen in recreational waters and the primary cause of hot tub folliculitis and otitis externa. The aim of this surveillance study was to determine the background prevalence and antimicrobial resistance profile of *P. aeruginosa* in swimming pools and hot tubs. A convenience sample of 108 samples was obtained from three hot tubs and eight indoor swimming pools. Water and swab samples were processed using membrane filtration, followed by confirmation with polymerase chain reaction. Twenty-three samples (21%) were positive for *P. aeruginosa*, and 23 isolates underwent susceptibility testing using the microdilution method. Resistance was noted to several antibiotic agents, including amikacin (intermediate), aztreonam, ceftriaxone, gentamicin, imipenem, meropenem (intermediate), ticarcillin/clavulanic acid, tobramycin (intermediate), and trimethoprim/sulfamethoxazole. The results of this surveillance study indicate that 96% of *P. aeruginosa* isolates tested from swimming pools and hot tubs were multidrug resistant. These results may have important implications for cystic fibrosis patients and other immune-suppressed individuals, for whom infection with multidrug-resistant *P. aeruginosa* would have greater impact. Our results underlie the importance of rigorous facility maintenance, and provide prevalence data on the occurrence of antimicrobial resistant strains of this important recreational water-associated and nosocomial pathogen.

## 1. Introduction

*Pseudomonas aeruginosa*, an important Gram-negative opportunistic pathogen, is the primary cause of hot tub folliculitis, otitis externa, as well as the principal cause of morbidity and mortality in cystic fibrosis patients [1]. *P. aeruginosa* is highly ubiquitous in water systems, and has intrinsic antimicrobial resistance due to low outer membrane permeability, as well as an extensive efflux pump system [2–4]. Additionally, some *P. aeruginosa* strains exhibit mutations in fluoroquinolone binding sites, the loss of porin channels, and increased beta-lactamase or cephalosporinase production [3,4]. *P. aeruginosa* frequently acquires additional resistance mechanisms (*i.e.*, from plasmids) and routinely develops multidrug resistance throughout the course of a treatment regimen [4,5]. The overall prevalence of antibiotic resistant *P. aeruginosa* is increasing, with up to 10% of global isolates found to be multidrug-resistant [6]. This represents a major treatment challenge, as *P. aeruginosa* is the second leading cause of gram-negative nosocomial infections [7].

Indoor recreational water is an important reservoir for *P. aeruginosa* and is a meaningful exposure pathway for bacterial transmission, where wet skin and occlusion provide optimal conditions for *P. aeruginosa* to thrive [8]. *P. aeruginosa* has been implicated in numerous nosocomial and community outbreaks, with therapy tanks and whirlpools frequently acting as the environmental reservoir [8–10]. Complex or hard to clean piping has been noted as a factor in *P. aeruginosa* contamination; in nosocomial and household settings, contaminated sinks and shower heads have been a common reservoir for *P. aeruginosa*, where the inaccessible armature is nearly impossible to adequately decontaminate [8,11]. Swimming pools and hot tubs have even more complex piping systems than sinks and showers, thus increasing the difficulty of cleaning. The temperature range in indoor recreational water is ideal for *P. aeruginosa* proliferation, which routinely grows in water 4–42 °C [8,12,13]. Finally, the high temperature and considerable agitation/aeration of hot tub water may cause rapid dissipation of halogen levels, thus rendering chlorine-based disinfectants ineffective [14,15].

While conditions are ideal for *P. aeruginosa* contamination, the background prevalence of *P. aeruginosa* in indoor recreational water has not been well evaluated during non-outbreak periods, where adequate chlorine levels are expected to prevent contamination [15,16]. Determining the endemicity of *P. aeruginosa* in indoor swimming pools and hot tubs during non-outbreak periods may have important infection control and facility maintenance implications. Our first study goal was to examine the prevalence of *P. aeruginosa* contamination in both of these indoor recreational waters, through the collection of water and swab samples.

The second goal of the study was to evaluate the level of antimicrobial resistance among the *P. aeruginosa* isolates from swimming pools and hot tubs. The presence of multidrug-resistant *P. aeruginosa* in a recreational environment may be important for immune-suppressed or other at-risk individuals, for whom treatment difficulties have greater implications [1]. Indeed, infections with drug-resistant *P. aeruginosa* often result in increased cost of treatment, lengthy stay, and overall morbidity and mortality [7,17,18]. In addition, given the propensity of *P. aeruginosa* to proliferate rapidly when disinfectant levels fall below recommended levels, monitoring the prevalence of resistant strains may be important for the prevention of future outbreaks.

## 2. Experimental Section

### 2.1. Sample Collection

A convenience sample of 50 water and 58 swab samples were obtained from eight indoor swimming pools and three hot tubs from March, 2009 through April, 2010 from throughout the central Ohio area in the United States. All swimming pools were public Class A or B competition or large recreation pools (Class A pools are large public pools intended primarily for competition; Class B pools are public recreation pools). One hot tub was a small residential model and two were public, large capacity systems. All public hot tubs and pools were operated by either an educational institution or municipality. Water samples were collected in sterile, polypropylene tubes, at approximately 0.30–0.50 meters below the water surface to best simulate an exposure zone; this depth is similar to a sampling regime recommended for beach water [19]. Sterile rayon swabs were used to collect biofilm from swimming pool and hot tub jets, strainers, and side drain locations. Swab samples were rotated (spun) both along the swab axis and moved laterally to collect an appropriate biofilm sample. All samples were transported to the laboratory on ice for immediate analysis. Aseptic technique was observed throughout sampling activities. Temperature readings were obtained while onsite using a digital thermometer.

Water samples were analyzed for free chlorine, total chlorine, and turbidity. Free and total chlorine levels were measured immediately according to DPD Method 10069 and 10070 using a Hach 2800 spectrophotometer (Hach, Loveland, CO, USA). The method reporting range is 0.1–10.0 mg/L (although >10.0 mg/L is provided by the Hach). Turbidity readings were measured using a Hach 2100P portable turbidimeter (Hach, Loveland, CO, USA).

### 2.2. Bacterial Isolation and Confirmation with PCR

Water samples were concentrated by membrane filtration using a sterile 0.45-μm-pore-size, 47-mm-diameter cellulose membrane filter (Millipore, Bedford, MA, USA) [19]. Swab samples were processed similarly, also using membrane filtration. In order to maximize bacterial recovery from a swab, the swab-containing tube was filled with approximately 5mL of buffer, vortexed for 10s, and the buffer passed through a 0.45-μm-pore-size, 47-mm-diameter cellulose membrane filter. All membrane filters were placed directly onto Pseudomonas Isolation Agar (Becton Dickinson, Sparks, MD, USA) and incubated at 37 °C for up to 48 hours. After incubation, up to three randomly selected colonies with appropriate morphological characteristics were sub-cultured for enrichment and purification using R2A agar with 0.5% yeast extract (in order to maximize the growth of the bacteria isolated from disinfectant-treated source). R2A plates were incubated at 37 °C for up to 48 hours. R2A was used because recovered bacteria may have been injured or stressed due to the presence of chlorine, and R2A is an efficient medium for recovering bacteria from disinfectant-containing samples by providing a low concentration of diverse nutrients [20]. In previous work, we modified this method by adding a low concentration of yeast extract, which showed good recovery of bacterial isolates from potable water [21]. Up to three isolates were obtained from purified, suspected *P. aeruginosa* samples, and confirmed using polymerase chain reaction (end-point and real time qPCR). Negative and positive controls (ATCC 27853) were utilized for all reactions. All end-point PCR was conducted using a MultiGene Thermal Cycler (Labnet International Inc., Edison, NJ, USA), under the following conditions: 95 ºC for 1 min; 40 cycles of denaturation at 95 ºC for 15 s, annealing at 58 ºC for 20 s; final extension at 68 ºC for 40 s; with primer pa722F (5′-GGCGTGGGTGTGGAAGTC-3′) and pa899R (5′-TGGTGGCGATCTTGAAC TTCTT-3′). For the real-time PCR, we used ABI Pseudomonas detection kit (Applied Biosystems, Foster City, CA, USA) with a 48-well StepOne™ Real Time System (Applied Biosystems, Foster City, CA, USA) following the manufacturer’s instructions.

### 2.3. Antimicrobial Susceptibility Testing

The minimum inhibitory concentration (MIC) to 22 antimicrobial agents was determined using a TREK Sensititre Sensitouch microdilution system (TREK, Cleveland, OH, USA). Twenty-three randomly selected isolates (at least one per *P. aeruginosa*-positive sample) were chosen for analysis and processed according to the manufacturer’s recommended protocol and the Clinical and Laboratory Standards Institute methods [22]. The 23 screened isolates represent 20 of the 23 total *P. aeruginosa*-positive samples; isolates from three samples failed to be subcultured sufficiently for microdilution analysis.

## 3. Results and Discussion

A total of 108 water (swimming pool samples n = 41; hot tub samples n = 17) and swab samples (swimming pool samples n = 36; hot tub samples n = 14) were collected from eight swimming pools and three hot tubs. Twenty-three samples (21%) were positive for *P. aeruginosa*, of which 16 were from hot tubs (70%); all samples from the residential hot tub were positive. Sixty-seven percent (2/3) of hot tubs and 63% (5/8) of swimming pools were positive at least once for *P. aeruginosa. P. aeruginosa*-positive samples were much more likely to come from swabs than from water samples. The positive swab locations were diverse, including side-wall tiles, gutter drains, jets, and strainer baskets which may indicate that these are biofilm-rich locations. Table 1 shows the prevalence data.

Water analysis indicated low turbidity overall (0.1–0.6 NTUs), and a wide range of free (FC) and total chlorine (TC) levels (FC: 0.8 to >10.0 mg/L, average 8.46 mg/L; TC: 1.0 to >10.0 mg/L, average >10 mg/L). Swimming pool temperature ranged from 25.9 °C to 32.4 °C, and hot tub temperature 38.7 °C to 39.3 °C. Differences were noted between *P. aeruginosa*-positive and negative samples with respect to water temperature and free chlorine. Turbidity levels were very similar between the groups. Water quality data were not available for the residential hot tub (which comprised 63% of the *P. aeruginosa* positive hot tub samples).

All 23 isolates analyzed were resistant to at least one antimicrobial, and several were multidrug-resistant. Of the clinically relevant antibiotics included in the screening panel, there was resistance to amikacin (intermediate, 9%), aztreonam (22%), ceftriaxone (4% resistant, 30% intermediate), gentamicin (9%), imipenem (26%), meropenem (intermediate, 4%), ticarcillin/clavulanic acid (4%), tobramycin (intermediate, 9%), and trimethoprim/sulfamethoxazole (13% resistant, 17% intermediate). Table 2 shows minimum inhibitory concentrations (MIC) to these antibiotics. High levels of resistance were noted to eight additional antibiotics (e.g., ampicillin, nitrofurantoin, *etc.*; See Table 3), but these were not included in the MIC analysis as they are not clinically indicated for the treatment *Pseudomonas aeruginosa* infections. Resistance is defined as an MIC result above the susceptibility upper bound, and intermediate resistance is defined as an MIC result above the susceptibility lower bound, but below the susceptibility upper bound.

### Discussion

The findings of this study indicate that *P. aeruginosa* contamination is common in swimming pools and hot tubs, even where chlorine concentrations are well above recommended levels. Recommendations for adequate chlorine levels are 2 to 4 mg/L for swimming pools, and 3 to 5 mg/L for hot tubs, measured as free chlorine [23]. According to NSPF recommendations, facilities should consider immediate closure when free chlorine levels fall below 1 mg/L, because contamination can quickly reach unsafe concentrations. With regard to hot tubs, prior research has demonstrated that even when hot tubs are drained, cleaned, and refilled, *P. aeruginosa* may return with a level of 10^4^–10^6^ cells/mL within 24 to 48 hours when adequate chlorine levels are not maintained [15]. In the current study, chlorine concentrations were higher than in past studies; and while *P. aeruginosa* was routinely isolated, the regularly high chlorine levels found here likely reduced prevalence. This confirms the importance of not only adequate disinfectant levels, but also constant monitoring and adjustment to ensure chlorine levels never drop below recommended levels. It should be noted that the facilities in this study conducted hourly monitoring of pool and hot tub chlorine concentrations, except the residence, which performed weekly monitoring. The less frequent monitoring of the residential hot tub may have resulted in greater halogen fluctuations, and thus higher prevalence of *P. aeruginosa*.

Consistent with previous research, a relationship between high chlorine levels, biofilm growth (as indicated by swab samples, which contain higher levels of biofilm-embedded bacteria, *versus* water samples, which are more likely to represent planktonic bacteria), and the presence of *P. aeruginosa*, was noted. LeChevallier *et al.* (1988) have described several mechanisms leading to disinfectant resistance, of which attachment to surfaces was most important [24]. They note that low-nutrient environments enhanced free chlorine resistance by two to three times. Thus, it is not surprising that this study indicated higher prevalence of *P. aeruginosa* in pools/hot tubs with higher chlorine levels. There was also a relationship between higher water temperature and the presence of *P. aeruginosa*. This finding is consistent with the thermo-tolerant nature of *P. aeruginosa*.

There are several possible reasons why *P. aeruginosa* was more likely to be isolated from hot tubs over swimming pools. Hot tub piping systems are complex and inaccessible for cleaning; this complexity may enhance biofilm formation [9]. In addition, hot tub capacity is lower (per bather) than swimming pools (0.757 m^3^/person for a hot tub *versus* 6.81 m^3^/person for swimming pools); this may act to concentrate microbial load present in the water [23]. Finally, high pressure jets are an integral component of hot tubs, with bathers often using jets to enhance relaxation. Contact with jets in this manner may enhance sloughing of skin squames, further fouling hot tub piping systems.

The results of this study also indicated that environmental *P. aeruginosa* has considerable levels of antibiotic resistance. Isolates demonstrated resistance to a wide range of clinically relevant antimicrobial agents, including antipseudomonal agents aztreonam (22%), gentamicin (9%), and imipenem (26%); and intermediate resistance to amikacin (9%), meropenem (4%), and tobramycin (9%). Resistance was also noted to alternate agents ceftriaxone (4% resistant, 30% intermediate), ticarcillin/clavulanic acid (4%), and trimethoprim/sulfamethoxazole (13% resistant, 17% intermediate). Although *P. aeruginosa* is a model of antimicrobial resistance (due to a number of intrinsic factors), past research has found that the degree of resistance to antipseudomonal agents varies considerably. The current results are similar to past studies, which have found analogous prevalence levels of resistant *P. aeruginosa* in both the hospital and community [4,10].

The origins of multidrug-resistant strains in the swimming pool and hot tub environment are unknown, but may have developed through several mechanisms. Since *P. aeruginosa* is intrinsically resistant, it is possible that the frequency with which we isolated resistant *P. aeruginosa* is representative of natural background levels. Alternately, in some cases resistant *P. aeruginosa* strains may ultimately be traced to a human source, either direct shedding from colonized humans or indirect transmission through the contamination in upstream aquatic reservoir. *P. aeruginosa* is not a predominant component of normal human flora (colonization rates are low: up to 2% on the skin and to 24% in fecal matter) [4,25]. However, during hospitalization, colonization rate may reach 50% [4,25]. Also, immune suppressed individuals or those whom have recently undergone antimicrobial treatment may have higher colonization rates [4,25,26]. In addition, in clinical settings, there is a strong relationship between the *P. aeruginosa* strains which infect patients and those in the immediate patient environment. Rogues *et al.* [27] pointed-out that carriage of *P. aeruginosa* by patients was “both the source and the consequence of (tap) water colonization”. That is, a colonized or infected patient may lead to in-room and neighboring area contamination; and contaminated water, fomites, and aerosols may infect susceptible individuals [27]. A similar phenomenon may occur in recreational water, particularly if frequented by colonized or infected individuals.

In some cases, there may be selective pressure for resistance by the presence of low-level antibiotic concentrations in either sweat secretions, which may enter recreational water through bather use, or from supply water. Prior research has demonstrated the presence of a range of antimicrobial agents in arm and axilla sweat secretions after oral or intravenous dosing [28]. While isolated concentrations were found to be quite low, this is a possible mechanism for the development of resistant strains. Antibiotics have also been routinely isolated in water at various stages of processing, including in post-chlorination drinking water [29]. Importantly, in some cases chlorinated water was found to select for more multidrug-resistant strains over non-chlorinated water [30]. Given that the swimming pool and hot tub supply water in our study begins as municipal drinking water but then becomes highly chlorinated to reach disinfection levels, there may be enhanced selection for resistant strains.

While the levels of resistance among study isolates are similar to those previously reported, it is important to draw the distinction between a nosocomial setting and the non-clinical, non-outbreak, recreational setting of the current study. In clinical scenarios, there is constant selective pressure to enhance the proliferation of multidrug-resistant strains. Given that *P. aeruginosa* has both intrinsic resistance and a dynamic ability to develop resistance during the course of infection, a high frequency of resistance is now expected in hospitals. However, in the non-clinical environment, the absence of selective pressure may reduce antimicrobial resistance levels. The presence of resistance to front-line antipseudomonal drugs may have important clinical and prevention implications. Given how common swimming pool and hot tub use is, immune-suppressed or other high-risk individuals may wish to exercise caution before use, especially hot tub use. Exposure to resistant *P. aeruginosa* results in even greater risk for individuals with cystic fibrosis.

Study limitations include a relatively small sample size and the convenience design. Although only one residential hot tub was accessible for sampling in this study, there was a desire to broadly evaluate whether differences existed in the prevalence of *P. aeruginosa* and the degree of antimicrobial resistance. The residential hot tub owner did not experience previous skin diseases related to the hot tub and the hot tub did not undergo thorough maintenance and flush-fill cycles as did the public hot tubs, which were cleaned daily, drained completely once per week, and have fresh water introduced continually. Further study is required with expanded sample size to determine if the frequency of multidrug-resistant strains remains steady over time. Additional studies should focus primarily on hot tubs, where 53% of samples were positive. Also, isolates from three *P. aeruginosa*-positive samples were unable to be revived for antimicrobial resistance analysis.

## 4. Conclusions

The results of this study indicate that *Pseudomonas aeruginosa* can be regularly isolated from both swimming pools and hot tubs, with substantial prevalence. In addition, isolated *P. aeruginosa* is resistant to a wide variety of antimicrobial agents, including front-line antipseudomonal drugs. These results are despite the high disinfectant levels noted in water samples. We believe that these findings underlie the critical importance of regular, thorough, aggressive maintenance to ensure a clean, healthy swimming pool and hot tub environment. The results confirm that high chlorine levels are not alone sufficient to prevent *P. aeruginosa* growth, and that notable levels of antimicrobial resistant *P. aeruginosa* are endemic in both hot tubs and swimming pools.

## Figures and Tables

**Table 1.** Prevalence of *P. aeruginosa* in hot tubs and swimming pools.

**Location**	**Pool/Spa Size (m^3^)**	**Approximate Bather Load (per day)**	**Average Water Temperature (°C)**	**Average Free Chlorine (mg/L)**	**Average Total Chlorine (mg/L)**	**Turbidity (NTU)**	***P. aeruginosa*-positive (%) Water (n)**	***P. aeruginosa*-positive (%) Swab (n)**
Hot tub–Residential	∼1.80	∼1	Not Available	Not Available	Not Available	Not Available	**100%** (6/6)	**100%** (4/4)
Hot tub–Public 1	8,593	60	39.1	6.2	6.8	0.1	**0%** (0/4)	**0%** (0/5)
Hot tub–Public 2	14,237	50	38.9	>10	>10	0.2	**0%** (0/4)	**75%** (6/8)
Swimming Pool 1	2,130,187	50	29.4	6.2	8.4	0.3	**0%** (0/4)	**25%** (1/4)
Swimming Pool 2	3,758,770	220	26.2	7.0	9.3	0.1	**0%** (0/4)	**25%** (1/4)
Swimming Pool 3	927,009	120	28.7	6.5	>10	0.3	**25%** (1/4)	**0%** (0/4)
Swimming Pool 4	590,997	110	27.4	7.8	>10	0.4	**0%** (0/4)	**0%** (0/4)
Swimming Pool 5	24,383	65	29.4	6.9	>10	0.2	**0%** (0/4)	**25%** (1/4)
Swimming Pool 6	Not Available	Not Available	26.7	5.1	5.5	0.4	**0%** (0/8)	**0%** (0/8)
Swimming Pool 7	Not Available	Not Available	28.6	9.8	>10	0.1	**0%** (0/4)	**0%** (0/4)
Swimming Pool 8	Not Available	Not Available	32.4	>10	>10	0.1	**0%** (0/4)	**33%** (3/9)

**Table 2.**
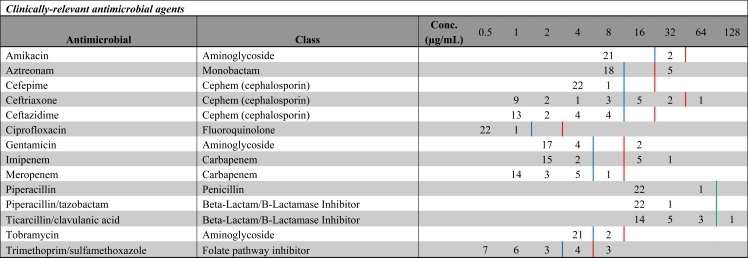
Minimum inhibitory concentrations (MICs) to 14 clinically-relevant antimicrobial agents for *P. aeruginosa*. Blue lines represent susceptibility lower bound and red lines represent susceptibility upper bound. Green lines indicate no intermediate susceptibility breakpoint zone.

**Table 3.** Percentage of resistance of *P. aeruginosa* isolates to eight additional antimicrobial agents.

***Additional antimicrobial agents tested***		

**Antimicrobial**	**Class**	**% Resistant**

Ampicillin	Penicillin	74
Ampicillin/sulbactam	Beta-Lactam/B-Lactamase Inhibitor	74
Cefazolin	Cephem (cephalosporin)	96
Cefotetan	Cephem (cephalosporin)	74
Cefoxitin	Cephem (cephalosporin)	78
Cefpodoxime	Cephem (cephalosporin)	30
Cefuroxime	Cephem (cephalosporin)	74
Nitrofurantoin	Nitrofurantoin	96
